# Data Augmentation for EEG-Based Emotion Recognition Using Generative Adversarial Networks

**DOI:** 10.3389/fncom.2021.723843

**Published:** 2021-12-09

**Authors:** Guangcheng Bao, Bin Yan, Li Tong, Jun Shu, Linyuan Wang, Kai Yang, Ying Zeng

**Affiliations:** ^1^Henan Key Laboratory of Imaging and Intelligent Processing, PLA Strategic Support Force Information Engineering University, Zhengzhou, China; ^2^Key Laboratory for NeuroInformation of Ministry of Education, School of Life Sciences and Technology, University of Electronic Science and Technology of China, Chengdu, China

**Keywords:** data augmentation, electroencephalography (EEG), emotion recognition, generative adversarial network (GAN), variational auto encoder (VAE)

## Abstract

One of the greatest limitations in the field of EEG-based emotion recognition is the lack of training samples, which makes it difficult to establish effective models for emotion recognition. Inspired by the excellent achievements of generative models in image processing, we propose a data augmentation model named VAE-D2GAN for EEG-based emotion recognition using a generative adversarial network. EEG features representing different emotions are extracted as topological maps of differential entropy (DE) under five classical frequency bands. The proposed model is designed to learn the distributions of these features for real EEG signals and generate artificial samples for training. The variational auto-encoder (VAE) architecture can learn the spatial distribution of the actual data through a latent vector, and is introduced into the dual discriminator GAN to improve the diversity of the generated artificial samples. To evaluate the performance of this model, we conduct a systematic test on two public emotion EEG datasets, the SEED and the SEED-IV. The obtained recognition accuracy of the method using data augmentation shows as 92.5 and 82.3%, respectively, on the SEED and SEED-IV datasets, which is 1.5 and 3.5% higher than that of methods without using data augmentation. The experimental results show that the artificial samples generated by our model can effectively enhance the performance of the EEG-based emotion recognition.

## Introduction

Affective computing refers to calculations related to emotion, generated from emotion, or influencing emotion (Tao and Tan, 2005). It has a wide range of applications in computer-aided learning, perceptual information retrieval, arts and entertainment, human health and interaction, wearable devices, and so on. Studies (Bocharov et al., 2017) have shown that mental diseases such as depression and autism are related to changes in emotional processing. Many methods have been devised for emotion recognition, which are mainly divided into two categories. One is the use of emotional behavioral features, such as facial expressions (Face Recognition and Emotion Recognition from Facial Expression Using Deep Learning Neural Network, 2020), body movements (Garber-Barron and Si, 2012), or voice (Bänziger et al., 2009) to identify human emotions. The other is the application of physiological signals to identify emotions, including ECG (Hsu et al., 2017), respiratory rate (Bloch et al., 1991), EMG (Mithbavkar, 2020), eye movement (Wang T. et al., 2020) and EEG. Compared with the former, physiological signals can produce more reliable recognition results. However, EEG signals have the advantages of high temporal resolution and recognition accuracy, and are considered to be one of the most reliable of physiological signals.

In recent years, an increasing number of researches have focused on deep learning in emotion recognition based on EEG (Ali et al., 2020; Jia et al., 2020; Li et al., 2020, 2021). However, they all ignored a key limitation: the lack of available EEG data. As generally known, deep neural networks, such as the classic image processing networks ResNet18 (He et al., 2016), Vgg16 (Simonyan and Zisserman, 2014), and AlexNet (Krizhevsky et al., 2017), require a great volume of data for training in order to obtain models with good performance. These all need a high data volume to train millions or even hundreds of millions of parameters. However, compared with image data and voice data, EEG data acquisition necessitates the use of expensive equipment, time, and manpower. These burdens all lead to the insufficiency of available EEG data volume. At present, the number of data in open image datasets, such as ImageNet (Krizhevsky et al., 2017) and CIFAR-10 (He et al., 2016), has reached tens of thousands or even tens of millions. In contrast, the public datasets of EEG emotion only include SEED (Zheng and Lu, 2015), DEAP (Koelstra et al., 2012) and MAHNOB-HCI (Soleymani, 2012), which are all much smaller. In addition, it is known that due to the non–stationarity of EEG signals, each subject and even each session will produce great variance (Lotte et al., 2007), resulting in the need to match each individual experiment. The matching process becomes difficult, as it needs to consider the differences between individual experiments, which will further affect the training process of the machine learning model. Moreover, the amount of data per subject is too small, therefore it is a great challenge to achieve the training of the applied deep neural network model.

One of the methods to solve the issue of data scarcity is to generate new data by transforming the original data, where the data distribution of the new data will be similar to that of the original data. This method is called data augmentation, which is generally divided into traditional methods and machine learning-based methods. Traditional data augmentation includes geometric transformation and noise addition. Compared with image processing, traditional methods are not friendly to EEG signals, because they are time series that cannot be translated, flipped, or rotated. If the EEG signal is noisy, the amplitude and data distribution of the original signal will be changed. In studies of emotion recognition based on EEG, some researchers first extract the features of EEG signals, and then add Gaussian noise to the features to generate new feature samples. The results demonstrate that the performance of the traditional classifier is hardly improved or even reduced by the expanded samples, while the performance of the deep neural network model is improved (Wang et al., 2018).

Data augmentation methods based on machine learning have become highly popular in recent years (Pascual et al., 2017; Wang et al., 2018; Gao et al., 2020), and include the generative adversarial network (GAN) (Goodfellow et al., 2014) and the variational auto-encoder (VAE) (Kingma and Welling, 2014). Since GAN can generate artificial data similar to the original data, many researchers use it to generate artificial images to expand the data and improve the recognition rate and stability of the image. Compared with the traditional methods, the data augmentation method based on GAN can generate more similar and more diverse data. Luo and Lu (2018) have done a lot of work on data augmentation for EEG-based emotion recognition. They proposed a method of generating EEG emotion samples based on conditional Wasserstein GAN (CWGAN) using the maximum mean discrepancy (MMD) to calculate the distribution distance between real samples and generated samples. In addition, the team also introduced conditional balanced GAN (cBEGAN) to generate EEG and eye movement feature samples for multimodal emotion recognition. Compared with Wasserstein GAN (WGAN), cBEGAN exhibits the advantages of stable training and fast convergence. The combination of EEG data and eye movement data can effectively improve the accuracy of emotion recognition (Luo et al., 2019). Considering that the quality of the generated samples will have great impact on the training of the model, they proposed a strategy of selecting samples using the SVM classifier to classify the generated samples. The samples with high classification confidence are considered as high-quality samples, and the samples with low classification confidence are excluded. The classification accuracy of data augmentation with a selection strategy is higher than that without a selection strategy.

The VAE is composed of an encoder and a decoder, whose purpose is to reconstruct the given data to generate new data. The encoder infers the variation of the original data and generates the variation probability distribution of the hidden variables; the decoder reconstructs the variation probability distribution of the hidden variables into the approximate probability distribution of the original data. The VAE is widely used in various fields. Yun et al. (2020) used conditional VAE (CVAE) to generate sufficient training data to solve the problem of metal surface defect classification. Wang Q. et al. (2020) proposed the norm-VAE to generate comprehensive features of the target domain and solve the unsupervised domain adaptation (UDA) problem in image classification. Aznan et al. (2019) used VAE to generate EEG signals to solve the problem of insufficient data, and utilized the generated data to train the SSVEP classifier; the results showed that the synthetic data can effectively improve the classification performance. The VAE has received extensive attention in image generation, however, it has a serious disadvantage in that the generated image is often very fuzzy, and its expression ability is poor for complex images. Moreover, the data generated based on the traditional GAN will present the phenomenon of pattern collapse. This phenomenon entails that the data generated in the generator is highly similar to the real data, but its diversity is insufficient. There are many patterns in real data, and the generator can only generate several of them but not all, which causes the lack of diversity.

The VAE can establish the relationship between latent vector and real data through a decoder, thus its ability to analyze complex data is limited, resulting in a blurred image (Bao et al., 2017). The GAN can capture the global information of the data, but the training is unstable and prone to pattern collapse, leading to the insufficient diversity of the generated data. To date, many studies have attempted to overcome the above shortcomings by combining VAE and GAN. Bao et al. (2017) proposed the image generative model CVAE-GAN, which combines CVAE and GAN to model the natural image as a probability model composed of labels and latent vectors. The results showed that, compared with CVAE and GAN alone, the image generated by CVAE-GAN is more fine-grained and has better diversity. Ye and Bors (2020) proposed the Lifelong VAEGAN (L-VAEGAN) data generative model, which can learn the information of latent variables over time and generate higher-quality samples. At the same time, it can autonomously learn the shared latent variables in different fields and realize cross domain reasoning.

Inspired by VAE and GAN, we propose a novel model named VAE-D2GAN, which can give full play to the advantages of both VAE and GAN. The encoder of a VAE maps the actual data to the latent space of specific distribution, and inputs the latent vector with specific distribution into the generator to learn the distribution of actual data more accurately and efficiently. In addition, we add an extra discriminator to GAN, namely, a double discriminator. Its components are all composed of neural networks and have the same structure but they do not share the same parameters. The functions of these two discriminators are different; they are opposite. The first discriminator tends to give high scores to samples that match the real data distribution, and the other discriminator tends to give high scores to samples generated by the generator. As a result of the game between the generator and the two discriminators, the distribution of the artificial samples generated by the generator will be infinitely close to the distribution of real data, so as to effectively avoid the excessive concentration of the distribution of artificial samples, which would lead to pattern collapse.

In addition, we use a variety of evaluation algorithms to assess the quality of the generated feature samples, such as Inception Score (IS; Salimans et al., 2016), Fréchet Inception Distance (FID; Heusel et al., 2017) and MMD (Borgwardt et al., 2006), so that the robust evaluation of the performance of the data augmentation model can be performed.

The main contributions of this paper are as follows:

1) Aimed at the problem of data scarcity in EEG emotion recognition, we propose a novel data augmentation model called VAE-D2GAN, which consists of an encoder, a shared decoder or generator, and two opposing discriminators. The first two networks aim to learn the spatial relationship of topological graph. The effect of the two discriminators is opposite: the first discriminator tends to the real data distribution, and the other discriminator tends to the generated data distribution. The samples generated by the generator deceive the two discriminators, so as to effectively avoid the problem of mode collapse. Once trained, the combined VAE-D2GAN can generate diverse artificial samples to enhance the classification model.
2) We conduct emotion classification experiments on two public datasets (SEED and SEED-IV). The results show that the artificial samples generated by our model can effectively augment the deep classification network, and the augmentation is enhanced in comparison to the current popular models, such as VAE, WGAN, and DCGAN.

## Methods

In this section, we propose a data augmentation model for EEG-based emotion recognition using Variational Auto Encoder and Generative Adversarial Network, which is called VAE-D2GAN. In this model, the spatial distribution of actual data is learnt from latent vectors through VAE, and the generator and the dual discriminator in GAN are then used to generate high-quality artificial samples that are similar to real samples but show an extent of diversity. Since GAN and VAE are both sensitive to image generation, emotion features are extracted from EEG signals as images to act as the input of the model.

### VAE-D2GAN Model

**FIGURE 1:**
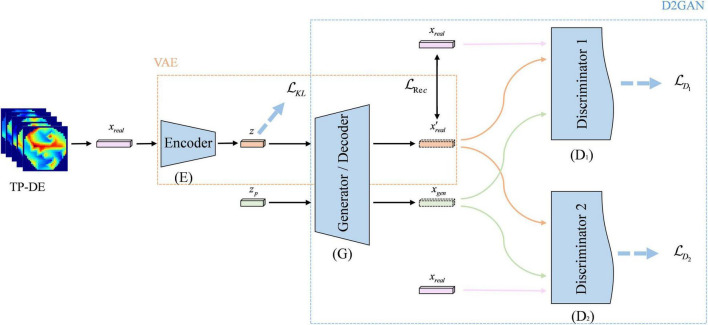
The framework of VAE-D2GAN. The model consists of an encoder, a decoder/generator, and two discriminators.

The framework of our proposed VAE-D2GAN model is shown in Figure 1. It consists of four parts: encoder (E); decoder or generator (G); discriminator 1 (D_1_); discriminator 2 (D_2_). The encoder E and generator G constitute the VAE. The real samples are mapped to latent vectors by the encoder E, and the latent vectors then generate the artificial samples by the decoder G. Generator G and discriminators D_1_ and D_2_ constitute the GAN. Generator G learns the real data distribution through the gradient calculated by the discriminators D_1_ and D_2_. D_1_ and D_2_ are dedicated to distinguishing real samples from generated samples.

#### Feature Extraction

Since Differential Entropy (DE) is the most widely used feature in the field of EEG-based emotion recognition (Wang et al., 2019; Zhang et al., 2019; Zhong et al., 2020; Cao et al., 2021; Liang et al., 2021) and our proposed model is more efficient at handling images, DE topological maps were extracted from the EEG signals as emotion features to act as the real sample input for the data augmentation model VAE-D2GAN.

Short-time Fourier transform was used to transform each segment of data. The DE feature can be expressed by the following formula:


(1) $$\begin{array}{c} h\,\left(X\right)=-\int_{\infty }^{\infty }\frac{1}{\sqrt{2\,\pi \,\sigma^{2}}}\,e^{-\frac{{\left(x-\mu \right)}^{2}}{2\,\sigma^{2}}}\,\log\left(\frac{1}{\sqrt{2\,\pi \,\sigma^{2}}}\,e^{-\frac{{\left(x-\mu \right)}^{2}}{2\,\sigma^{2}}}\right)\,dx\\ =\frac{1}{2}\,\log\left(2\,\pi \,e\,\sigma^{2}\right) \end{array}$$

where *X* follows a Gaussian distribution *N*(μ,σ^2^), *x* is a variable, and *e* and π are constants.

**FIGURE 2:**
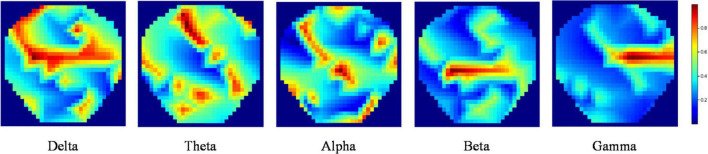
TP-DE images of five frequency bands for a certain participant. The length and width are 32; the channel is 5.

We calculated the DE feature of five bands for each channel, Delta band (1–4 Hz), Theta band (4–8 Hz), Alpha (8–14 Hz), Beta band (14–31 Hz), and Gamma band (31–50 Hz). The linear dynamic system method was used to filter the noises and artifacts that were unrelated to EEG features (Shi and Lu, 2010). Next, the DE features were transformed into 32*32*5 (length and width = 32, channel = 5) topology images according to the method in the literature (Bao et al., 2021), namely TP-DE, as shown in Figure 2.

The strategy of transforming the traditional DE features into topological maps with image characteristics and using them to be the real sample input can better match the network structure of the proposed augmentation model. Moreover, the topological map features are more explanatory and convenient for judging the quality of the artificial samples.

#### VAE

In this section, we introduce the structure and function of VAE (Kingma and Welling, 2014). In the proposed VAE-D2GAN model, VAE learns the latent information from real samples through the encoder and the decoder.

The TP-DE topological maps extracted from real EEG data were extended into one-dimensional feature vectors x_real_ as input for VAE. Input *x*_real_ into encoder E to return the estimation of posterior data distribution *q*(*z*|*x*_real_), input the low dimensional latent vector *z* into decoder G, and reconstruct conditional distribution *p*(*x*_real_|*z*) of data under the constraint of prior distribution *p*(*z*), where *q*(*z*|*x*_real_) and *p*(*x*_real_|*z*) are usually represented as follows:


(2) $$z∼E\,\left(x_{r\,e\,a\,l}\right)=q\,\left(z|x_{r\,e\,a\,l}\right),x_{r\,e\,a\,l}^{\prime }∼G\,\left(z\right)=p\,\left(x_{r\,e\,a\,l}|z\right)$$

where *E*(⋅) represents the encoder, *G*(⋅) represents the decoder (or generator), and $x_{r\,e\,a\,l}^{\prime }$ represents the reconstruction samples.

The latent vector *z* is a combination of the mean value μ and the standard deviation σ output by the encoder E, and is expressed as follows:


(3) z=μ+γ⊙exp⁡(σ)

where γ∼*N*(0,I) is a random vector which obeys Gaussian distribution, ⊙ denotes multiplication by elements, therefore we assume that the latent vector *z* approximately confirms to Gaussian distribution *z*∼*N*(μ,exp(σ)^2^).

The Kullback-Leibler (KL) divergence is introduced to optimize the parameters of the encoder, and the KL divergence loss formula is as follows:


(4) $${\text{L}}_{K\,L}=KL\left(q\left(z|x_{r\,e\,a\,l}\right)||p\left(z\right)\right)$$

where *KL*(⋅) represents the calculation of KL divergence distance.

Besides the KL divergence loss, VAE also uses reconstruction loss to optimize the decoder, and the formula of reconstruction loss is as follows:


(5) $${\text{L}}_{R\,e\,c}=-{\text{E}}_{q\,\left(z|x_{r\,e\,a\,l}\right)}\,\left[\log p\,\left(x_{r\,e\,a\,l}|z\right)\right]$$

Formula 5 calculates the square of Euclidean distance between the real data and the synthetic data.

Therefore, the total loss of VAE is expressed as:


(6) $${\text{L}}_{V\,A\,E}={\text{L}}_{K\,L}+{\text{L}}_{R\,e\,c}$$

#### D2GAN

The D2GAN model (Nguyen et al., 2017) is introduced into the proposed VAE-D2GAN model to ensure the diversity of the generated samples.

Differently from the traditional GAN, D2GAN consists of a generator G and two discriminators D_1_ and D_2_. D_1_ gives preference to the samples from real data and attributes them a high score. On the contrary, D_2_ prefers the samples generated by the generator and scores them high. The input of generator G is the random variable *z*_*p*_, which obeys the Gaussian distribution *z*_*p*_∼*N*(0,I). Moreover, the output is the generated sample *x*_*gen*_.

The expression of output *x*_*gen*_ is the following:


(7) $$x_{g\,e\,n}=G\,\left(z_{p}\right)$$

where *G*(⋅) represents the generator network.

In D2GAN, G, D_1_ and D_2_ play the following three-player game:


(8) $$\begin{array}{c} \min_{G}\max_{D_{1},D_{2}}\alpha \,\text{E}\,\left[\log D_{1}\,\left(x_{r\,e\,a\,l}\right)\right]+\text{E}\,\left[-D_{1}\,\left(G\,\left(z_{p}\right)\right)\right]\\ +\text{E}\,\left[-D_{2}\,\left(x_{r\,e\,a\,l}\right)\right]+\beta \,\text{E}\,\left[\log D_{2}\,\left(G\,\left(z_{p}\right)\right)\right] \end{array}$$

where *D*_1_(⋅) and *D*_2_(⋅) represent discriminator 1 and discriminator 2, respectively. Their network structures are the same, but their parameters are not shared. The parameters α and β are hyperparameters, which follow 0 < α,β≤1.

#### VAE-D2GAN

Since the image generated by the traditional VAE is fuzzy (Bao et al., 2017), the representation ability of the generated images is weak. The D2GAN is introduced into the model to try to learn complementary information while avoiding the pattern collapse problem caused by GAN.

With VAE combined into the model, the loss for generator G in D2GAN is expressed as:


(9) $${\text{L}}_{G}=-{\text{L}}_{G\,D_{1}}+\beta \,{\text{L}}_{G\,D_{2}}$$

(10) $${\text{L}}_{G\,D_{1}}=\text{E}\,\left[D_{1}\,\left(G\,\left(z\right)\right)\right]+\text{E}\,\left[D_{1}\,\left(G\,\left(z_{p}\right)\right)\right]$$

(11) $${\text{L}}_{G\,D_{2}}=\text{E}\,\left[\log D_{2}\,\left(G\,\left(z\right)\right)\right]+\text{E}\,\left[\log D_{2}\,\left(G\,\left(z_{p}\right)\right)\right]$$

where β is the hyperparameter, which is the same as that in Formulas 8. *D*_1_(⋅) and *D*_2_(⋅) represent discriminator 1 and discriminator 2, respectively. Their network structures are the same, but their parameters are not shared. We optimize the generator by minimizing its loss.

Since there are two independent discriminators in D2GAN, two different loss optimization discriminators are also needed:


(12) $${\text{L}}_{D_{1}}=\alpha \,\text{E}\,\left[\log D_{1}\,\left(x_{r\,e\,a\,l}\right)\right]+\text{E}\,\left[-D_{1}\,\left(G\,\left(z\right)\right)\right]+\text{E}\,\left[-D_{1}\,\left(G\,\left(z_{p}\right)\right)\right]$$

(13) $${\text{L}}_{D_{2}}=\beta \,\text{E}\,\left[\log D_{2}\,\left(G\,\left(z_{p}\right)\right)\right]+\beta \,\text{E}\,\left[\log D_{2}\,\left(G\,\left(z\right)\right)\right]+\text{E}\,\left[-D_{2}\,\left(x_{r\,e\,a\,l}\right)\right]$$

where α and β are hyperparameters, which are the same as those in Formulas 8. The reason for the introduction of hyperparameters α and β is to make the training more stable by adjusting the penalty of D_1_ and D_2_.

Subsequently, VAE-D2GAN becomes a four-player game optimized by E, G, D_1_ and D_2_:


(14) $$\min_{E,G}\max_{D_{1},D_{2}}\text{L}\,\left(E,G,D_{1},D_{2}\right)={\text{L}}_{K\,L}+{\text{L}}_{R\,e\,c}+{\text{L}}_{G}+{\text{L}}_{D_{1}}+{\text{L}}_{D_{2}}$$

L_*KL*_ is only related to the encoder. Similarly, L_*D*_1__ and L_*D*_2__ correspond to the target losses of D_1_ and D_2_, respectively. Since VAE and D2GAN share the generator, the loss of generator consists of two parts: L_*G*_ and L_*Rec*_, where L_*G*_ is the loss of D2GAN and L_*Rec*_ is the loss of VAE. The specific training process is shown in Algorithm 1.

**ALGORITHM 1:** The training process of VAE-D2GAN.

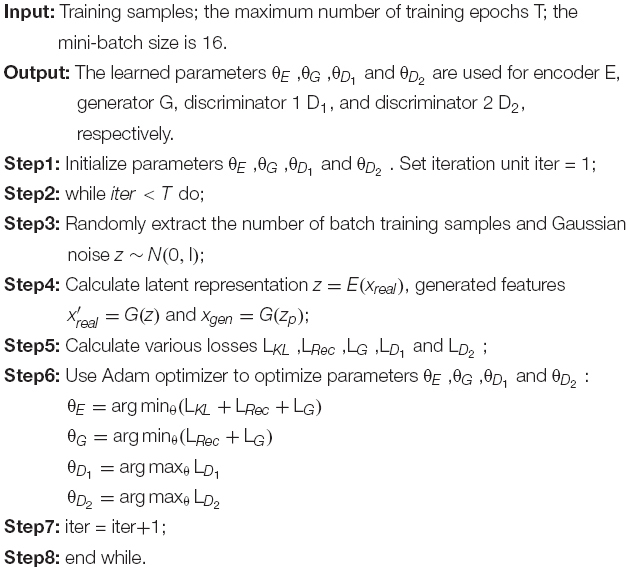

After training the whole model, the Gaussian noise is passed through the trained generator to generate high-quality samples.

### Classifier Based on Deep Neural Network

**FIGURE 3:**
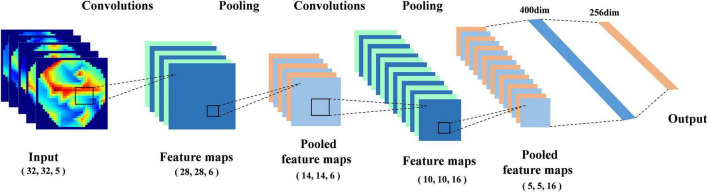
Structural diagram of deep neural network (DNN).

In our previous work (Bao et al., 2021), we proposed a deep neural network (DNN) classification model that can effectively extract the features of the topological graph; its structure is shown in Figure 3. We add the AdaBN layer (Li et al., 2018) after each convolution layer and full connection layer, which standardizes the distribution between the real samples and the generated samples in each batch. In order to compare the performance of different classification models, we compare the classical networks in the field of image processing, such as RestNet18, VGG16 and AlexNet, etc.

**FIGURE 4:**
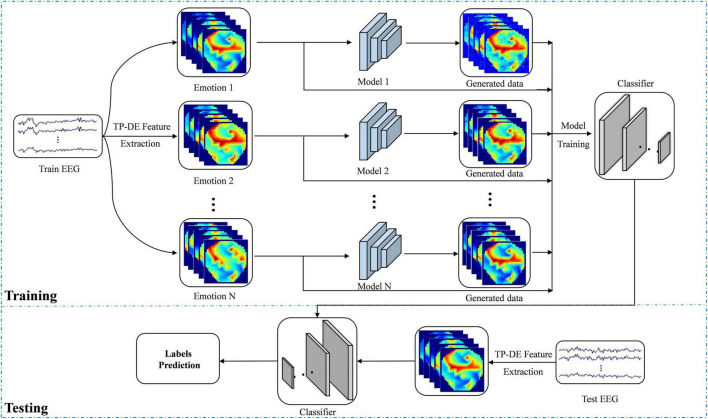
Flowchart of data augmentation based on emotion recognition.

In addition, we propose a sample data augmentation strategy that, according to each type of emotion training their own data augmentation model, can better learn the characteristics of the same emotion. The overall flow of data augmentation is shown in Figure 4. We transform the EEG signal into feature image as the input of the data augmentation model. There is one data augmentation model for each kind of emotion training, and there are N data augmentation models for N kinds of emotion. The structure of each model is consistent but the parameters are independent. Finally, the generated samples and real samples are used as the training set to train the deep neural network in order to classify the testing set.

### Evaluate the Quality of Generated Samples

Evaluating the quality of generated samples is one of the methods to verify the effectiveness of the data enhancement model. In the field of image processing, IS and FID are two common indicators to evaluate the quality of generated images. The samples generated in this paper are topology images, so we also use IS and FID to evaluate them. In addition, MMD algorithm is used to calculate the distribution distance between real samples and generated samples to understand the distribution of generated samples.

Inception Score is a common method to evaluate the performance of a data augmentation model. It uses a fixed classifier to predict the generated image, and obtains the conditional entropy of the prediction label. The larger the conditional entropy, the higher the quality of the generated image will be. At the same time, the edge probability is used to judge the diversity of the generated image. The higher the edge probability, the greater the diversity will be. The IS combines conditional entropy and edge probability; the larger it is, the better quality and diversity of samples will be generated. Nonetheless, it has a fatal flaw in that when the pattern collapses, the IS value will be fairly high. In this paper, we employ the DNN network as the initial classification model. We use all data in each dataset to train a general basic model, and replace Inception v3 with this trained model.

Fréchet Inception Distance is a further improved method to evaluate the performance of the data augmentation model. Like IS, FID needs a fixed classifier to classify samples. As opposed to IS, however, FID introduces real samples, extracts the features of real samples and generated samples in the middle layer of the classification model, and calculates the Wassertein-2 distance between the real samples and the generated samples. The smaller the FID, the higher sample quality and diversity can be attained. Compared with IS, FID is more sensitive to pattern collapse and is more stable against noise.

Maximum mean discrepancy is often used to measure the distance between two distributions. Firstly, two distributions are mapped to the regenerative kernel Hilbert space (RKHS), and the distance after mapping is calculated. By calculating the MMD distance between the real samples and the generated samples, we can verify whether the samples generated by the generator and the real samples have the same distribution, that is, we can check the quality of the generated samples.

## Experimental Settings

### Datasets

In this section, we introduce the datasets used in this paper: SEED and SEED-IV.

At present, in the field of EEG emotion recognition, the SEED dataset (Zheng and Lu, 2015) constructed by the SJTU is one of the most widely used datasets. In this dataset, 15 healthy subjects (8 females and 7 males, mean: 23.27, SD: 2.37) were collected by the ESI Neuroscan System. The sampling rate was 1000 Hz and there are 62 electrode channels, which meets the international 10–20 standard. Scores (1–5) and keywords were used to evaluate the subjects’ three kinds of emotions (positive, neutral, and negative) while they were watching video clips. Each of 15 video clips lasted for about 4 min. Herein, the original EEG data are processed by a series of pretreatment, such as downsampling to 200 Hz, removing the signal seriously polluted by EOG and EMG, and then passing the selected signal through a 0.3–50 Hz band-pass filter. Finally, data are divided into 1 s data segments without overlapping, and each segment of data is taken as a sample. Therefore, each subject has a total of 3,394 samples, and the sample size of the three types of emotions is basically the same. Each subject participated in the experiment three times with an interval of 1 week. In this study, we use the EEG data of each subject for the first time.

The SEED-IV dataset (Zheng et al., 2018) selects 72 video clips to induce four different emotions (happy, neutral, sad, and fear), and each video clip lasts for about 2 min. Twenty-four trials (6 trials for each kind of emotion) were conducted in each experiment. Each subject participated in three experiments at different times. A total of 15 healthy subjects (8 females and 7 males) were recruited by the ESI Neuroscan System, 62 channels EEG data of the international 10–20 system were recorded with a sampling rate of 1000 Hz, and the eye movement signals were collected simultaneously. For the preprocessing of EEG signal, the original EEG signal is downsampled to 200 Hz, and the noise and artifact are eliminated by using a band-pass filter of 1–75 Hz. Each trial is divided into 4s data segments without overlapping, and each segment of data is taken as a sample. This operation results in 851 samples for each subject, and the sample size of four emotions is basically the same. In this study, we use the EEG data of each subject for the first time.

### Training Settings

Firstly, the structure details of encoder (E), generator (G) and two discriminators (D_1_, D_2_) in VAE-D2GAN proposed in this paper are introduced, as shown in Table 1. An Adam optimizer was used, and the learning rate was 0.0001. The batch size was 16. All the methods in this paper were implemented in Python, and the deep neural network was implemented in Tensorflow.

**TABLE 1:** VAE-D2GAN architecture.

Module	Layer	Kernel size	Stride	Input	Output	Activation
Encoder	Input	–	–	–	(*n*, 5,32,32)	–
	Conv1	(5,5)	2	(*n*, 5,32,32)	(*n*, 64,16,16)	ReLU
	Conv2	(5,5)	2	(*n*, 64,16,16)	(*n*, 128,8,8)	ReLU
	Conv3	(5,5)	2	(*n*, 128,8,8)	(*n*, 256,4,4)	ReLU
	FC1	–	–	(*n*, 256*4*4)	(*n*, 128)	–
Generator	Input	–	–	64	(*n*, 128)	–
	FC1	–	–	(*n*, 128)	(*n*, 256*4*4)	ReLU
	Deconv1	(5,5)	2	(*n*, 256,4,4)	(*n*, 128,8,8)	ReLU
	Deconv2	(5,5)	2	(*n*, 128,8,8)	(*n*, 64,16,16)	ReLU
	Deconv3	(5,5)	2	(*n*, 64,16,16)	(*n*, 5,32,32)	ReLU
Discriminator	Input	–	–	–	(*n*, 5,32,32)	–
	Conv1	(5,5)	2	(*n*, 5,32,32)	(*n*, 64,16,16)	ReLU
	Conv2	(5,5)	2	(*n*, 64,16,16)	(*n*, 128,8,8)	ReLU
	Conv3	(5,5)	2	(*n*, 128,8,8)	(*n*, 256,4,4)	ReLU
	FC1	–	–	(*n*, 256*4*4)	(*n*, 1)	–

The * represents the multiplication symbol.

Then, we utilize the experience of previous researchers on the training set and testing set of SEED and SEED-IV datasets. For the SEED data set, we take the data of the first nine sessions as the training set, and the last six sessions as the testing set, in which the last six sessions contain two sessions of positive, neutral, and negative emotions. In the same manner, for the SEED-IV data set, we take the data of the first 16 sessions as the training data, and the last 8 sessions as the testing set. Among them, the last 8 sessions include two sessions of happy, neutral, sad, and fear emotions.

In addition, we added extended experiments on the SEED dataset to explore the impact of different numbers of training samples on the performance of data enhancement model. The specific training settings are as follows:

Experiment 1: We select the first three sessions (positive, neutral and negative, each with one session) of each subject as the training set, and the last six sessions as the testing set.

Experiment 2: We select the first six sessions (positive, neutral and negative, each with two sessions) of each subject as the training set, and the last six sessions as the testing set.

Experiment 3: We select the first nine sessions (positive, neutral and negative, each with three sessions) of each subject as the training set, and the last six sessions as the testing set.

We use generative methods to generate artificial samples of all kinds of emotions. The SEED dataset includes three kinds of emotions (positive, neutral, and negative), and the SEED-IV dataset has four kinds of emotions (happy, neutral, sad, and fear). Each emotion type generates 8,000 samples. Therefore, on the SEED data, each subject generates 24,000 samples; on the SEED-IV data, each subject generates 32,000 samples.

## Results

### The Impact of Different Data Augmentation Models

In order to evaluate the performance of our proposed model in improving the accuracy of emotion recognition, we compare it with the current related data augmentation models. The results on the SEED and SEED-IV datasets are shown in Tables 2, 3, respectively. From Table 2, we can infer that the recognition accuracy of VAE and WGAN is not improved after using data augmentation, but is decreased. The DCGAN reaches its best mean accuracy of 91.6% when 15,000 artificial samples are added, the D2GAN reaches its best mean accuracy of 91.6% when 20,000 artificial samples are added, and the VAE-D2GAN reaches its best mean accuracy of 92.5% when 2,000 artificial samples are added. The accuracy of DCGAN, D2GAN, VAE-GAN, and VAE-D2GAN is 0.6, 0.6, 0.6, and 1.5% higher, respectively, than that without data augmentation. The VAE-D2GAN exhibits the best performance among all methods.

**TABLE 2:** On the SEED dataset, TP-DE images are generated based on different models, and the number of different generated images samples is added in the training set.

	Generated samples								
Model	0	1000	2000	5000	8000	10000	15000	20000	24000
VAE	91.0/7.2	88.7/8.5	89.4/7.8	90.7/7.5	89.6/6.6	88.5/7.6	89.0/7.9	90.4/6.8	88.9/7.9
WGAN	91.0/7.2	89.2/7.3	89.1/7.0	88.6/7.2	87.4/8.6	88.3/6.9	89.7/7.2	87.0/7.9	88.6/6.9
DCGAN	91.0/7.2	90.1/7.7	90.0/8.4	88.8/7.4	88.4/7.2	89.9/7.1	91.6/7.7	91.0/7.4	90.3/7.1
D2GAN	91.0/7.2	91.4/7.3	90.9/6.4	90.1/7.0	90.4/6.8	89.4/8.3	89.3/6.6	91.6/6.3	90.3/7.2
VAE-GAN	91.0/7.2	89.7/7.3	90.9/7.1	90.9/7.5	91.6/7.2	91.1/8.0	89.4/8.0	90.8/7.4	89.5/7.5
VAE-D2GAN	91.0/7.2	90.9/7.0	92.5/7.1	91.0/7.4	90.4/7.4	91.2/7.3	90.0/7.8	91.7/6.9	91.7/6.1

*The average accuracy and standard deviation of classification are obtained by using DNN. 0 means no generated samples are added in the training set.*

**TABLE 3:** On the SEED-IV dataset, TP-DE images are generated based on different models, and the number of different generated image samples is added in the training set.

	Generated samples										
Model	0	1000	2000	5000	8000	10000	15000	20000	25000	30000	32000
VAE	78.8/14.2	78.6/13.5	77.2/12.7	76.9/12.6	74.5/14.1	72.0/15.6	69.2/17.8	64.9/12.7	59.9/15.6	59.9/15.6	61.3/17.3
WGAN	78.8/14.2	76.1/13.5	78.0/10.3	71.5/11.3	73.7/13.7	71.2/14.1	68.6/13.9	69.4/14.2	65.6/14.4	67.7/13.6	70.4/14.3
DCGAN	78.8/14.2	76.8/12.2	72.2/12.4	78.1/11.6	76.9/13.8	75.6/10.8	76.5/10.1	77.4/11.8	77.5/11.6	79.1/13.8	79.1/11.7
D2GAN	78.8/14.2	78.3/11.9	80.0/12.6	73.8/14.0	76.2/13.5	75.0/12.4	75.4/14.3	76.3/13.5	75.1/11.3	74.3/12.6	75.1/12.7
VAE-GAN	78.8/14.2	78.8/11.2	80.8/10.3	77.7/89.2	78.9/9.6	81.1/11.5	80.7/11.0	80.8/11.8	81.5/12.8	81.1/12.9	80.5/12.5
VAE-D2GAN	78.8/14.2	78.8/11.4	80.4/10.6	80.8/12.3	81.4/11.4	82.3/11.0	79.9/13.0	79.0/10.5	80.2/11.6	80.5/11.8	80.8/10.1

*The average accuracy and standard deviation of classification are obtained by using DNN. 0 means no generated samples are added in the training set.*

Table 3 demonstrates that neither VAE nor WGAN improves the accuracy. The DCGAN reaches its best mean accuracy of 79.1% when 30,000 artificial samples are added, the D2GAN reaches its best mean accuracy of 80.0% when 2,000 artificial samples are added, and the VAE-D2GAN reaches its best mean accuracy of 82.3% when 10,000 artificial samples are added. The accuracy of DCGAN, D2GAN, VAE-GAN and VAE-D2GAN is 0.3, 1.2, 2.7, and 3.5% higher than that without data augmentation. The VAE-D2GAN has the best performance among all the methods. The extent of accuracy improvement of the SEED-IV dataset is higher than that of the SEED dataset. The reason for this phenomenon is that the number of samples in SEED-IV is far less than that in SEED. Therefore, the data augmentation effect for small volume sample data will be better.

**FIGURE 5:**
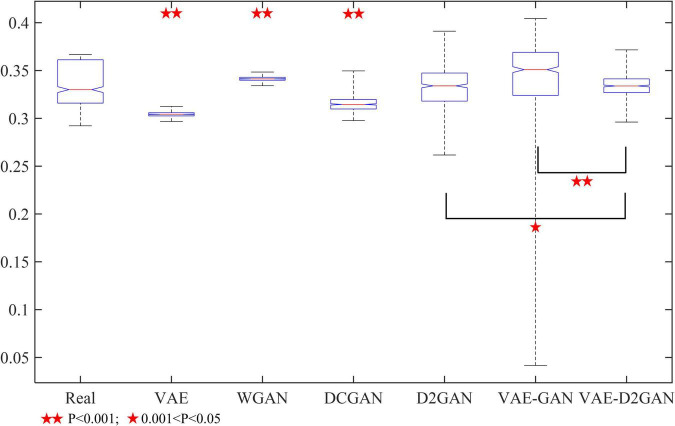
Significance test of different data enhancement models.

In order to further prove the effectiveness of our proposed model, we conducted a *t*-test to test the significance between different models. We randomly selected a certain number of synthetic samples for significance test. The results are shown in Figure 5. The samples synthesized from D2GAN, VAE-GAN and VAE-D2GAN were not significantly different from the actual samples (*P* > 0.05), which shows that these models can effectively learn the distribution of actual data. The synthetic data generated by VAE-D2GAN has the greatest correlation with the real data particularly (*P* = 0.9334). In addition, VAE-D2GAN was significantly different from D2GAN (*P* = 0.0139) and VAE-GAN (*P* = 0.0060), respectively.

### The Impact of Different Classifiers

**FIGURE 6:**
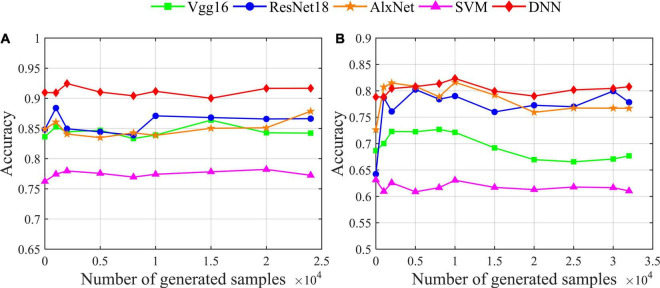
The influence of data augmentation on the recognition accuracy of different classifiers. **(A)** Result for the SEED dataset; **(B)** result for the SEED-IV dataset.

In this section, we use the proposed augmentation model for different classifiers (including various deep networks and traditional machine learning) to analyze the impact of recognition accuracy. From the deep network models, we choose the classic VGG16, ResNet18 and AlexNet; from the traditional machine learning models, we choose the classic support vector machine (SVM). At the same time, we use our data augmentation model VAE-D2GAN; the results are shown in Figure 6. The classification results obtained by different classifiers are different from those without data augmentation. On the SEED dataset, the accuracy of classification obtained by using Vgg16, ResNet18, AlexNet, SVM, and DNN classifiers is 83.64, 84.85, 84.89, 76.24 and 90.97%, respectively. On the SEED-IV dataset, the accuracy of classification obtained by using Vgg16, ResNet18, AlexNet, SVM, and DNN classifiers is 68.67, 64.23, 72.62, 63.13 and 78.83%, respectively. In general, deep networks are better than traditional machine learning methods. According to the results for the SEED and SEED-IV datasets, as shown in Figures 6A,B, respectively, DNN has the highest classification accuracy. In addition, data augmentation has little effect on traditional machine learning in EEG-based emotion recognition. Moreover, for deep networks, the effect of using data augmentation is enhanced, especially for small data sets.

In comparison with the more complex deep networks Vgg16, ResNet18, and AlexNet, DNN has a simple network structure but the best performance in identifying the topology images. Therefore, the higher complexity of network structure does not necessarily mean good classification performance. For the simple image classification of the topology, a simple network can get satisfactory classification performance. However, for more complex classification tasks, such as 4-classification on the SEED-IV data set (Figure 6B), the classification performances of ResNet18 and AlexNet are fairly close to that of DNN. Therefore, for a more complex classification task, a more complex network may have a better classification effect.

### The Quality of Samples Generated by Different Data Augmentation Models

In this section, we apply the IS, FID, and MMD algorithms in two datasets to evaluate the performance of our proposed model. The results are shown in Table 4, where the bold representation indicates the best results. Since the values of IS and FID in WGAN are higher than that of VAE-D2GAN, WGAN shows pattern collapse compared with VAE-D2GAN. The samples generated by VAE are of poor quality, leading to higher MMD and FID values. The samples generated by VAE-D2GAN are of high quality and good in diversity, since FID and MMD have the lowest values.

**TABLE 4:** Several algorithms are used to evaluate the performance of the data augmentation models.

	Evaluation method					
Model	SEED			SEED-IV		
	IS	FID	MMD	IS	FID	MMD
VAE	1.371	29.257	0.628	1.390	409.52	0.907
WGAN	**2.445**	17.511	0.175	**2.206**	67.906	0.347
DCGAN	1.874	30.108	0.171	1.566	40.122	0.508
D2GAN	1.951	20.745	0.111	1.845	13.762	0.241
VAE-GAN	2.256	17.557	0.241	1.995	30.542	0.276
VAE-D2GAN	2.041	**12.060**	**0.106**	1.865	**11.016**	**0.229**

*Bold represents the best performance in the corresponding evaluation algorithm.*

**FIGURE 7:**
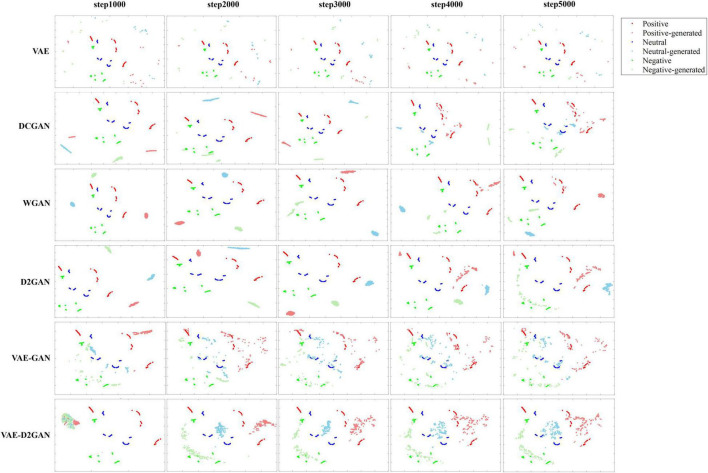
Two-dimensional visualizations of real and generated TP-DE images by different models at different iterations in the SEED dataset. Data points with red, blue and green represent real samples of positive, neutral and negative emotions, respectively, and the lighter color represents generated samples.

In order to better illustrate the advantages of VAE-D2GAN, we map the real samples and the samples generated by different models at different iterations to the two-dimensional visualizations through t-SNE, as shown in Figure 7. From the results we can observe that: (1) The model training effect of combining VAE and GAN is better than that of independent VAE and GAN, such as VAE-GAN and VAE-D2GAN. Because the encoder of VAE maps the actual data to the latent space of specific distribution, the generator can learn the distribution of actual data more accurately and quickly. (2) Compared with VAE-GAN, the sample distribution generated by VAE-D2GAN is more restrictive to prevent the distribution of synthetic data from being too scattered and affecting the recognition performance. It can also be seen from the results of FID and MMD algorithms that the performance of VAE-D2GAN is better than VAE-GAN.

### The Impact of Training Sample Quantity

In the previous section, we established that VAE-D2GAN is more friendly to small sample data volumes. Therefore, we carry out experiments on the recognition performance of different numbers of samples as the training set on the SEED data set.

The results are shown in Table 5. The recognition accuracy of Experiment 1, 2, and 3 using data augmentation is 79.46, 83.76 and 92.46%, respectively. Compared with no data augmentation applied, the accuracy is improved by 11.29, 8.3 and 1.49%, respectively. As the number of training samples from Experiment 1 to Experiment 3 are gradually increased, the recognition performance also gradually improves. However, the smaller the number of training samples, the higher the improvement of recognition performance.

**TABLE 5:** Three groups of experiments were set to explore the performance of the data augmentation model while varying the number of training samples for each experiment.

	Data augmentation	
Experiment	No	Yes
Experiment 1	68.17/11.89	79.46/12.24
Experiment 2	75.46/14.04	83.76/10.64
Experiment 3	90.97/7.20	92.46/7.05

*The average accuracy and standard deviation of classification were obtained by using DNN.*

## Conclusion

In this paper, we propose a data augmentation model named VAE-D2GAN for EEG-based emotion recognition. Through this model, we can better analyze the EEG emotion features, learn relevant specific spatial distribution from latent vectors, and combine two discriminators to generate more diverse samples. The proposed model is more stable in training on small sample dataset. Since the deep network is sensitive to images, we transformed the DE features of EEG signals into topological images by mapping and interpolation, and called this operation TP-DE. Not only it can convert an EEG signal into image form, but also retain the spatial information of the signal. We further conducted classification verification on two public emotional data sets, SEED and SEED-IV, with an accuracy rate of 92.5 and 82.3%, respectively. The accuracy of using the proposed data augmentation model was 1.5 and 3.5% higher than that without using one. Findings show that our data augmentation model can effectively enhance EEG signals for emotion recognition, and its performance is superior to that of VAE, WGAN, DCGAN, D2GAN, and VAE-GAN. Moreover, we explored the impact of the classification network compared with the classical deep networks Vgg16, ResNet18, AlexNet, and the traditional machine learning method SVM. The results show that the shallow network used to extract the features of simple images (such as TP-DE) exhibits superior performance. Hence, these results demonstrated that our model can effectively enhance the performance of the EEG-based emotion recognition. However, there are some low-quality samples in the data synthesized by the data augmentation model, which will reduce the recognition performance of classifier. Therefore, how to select high-quality samples from synthetic data is a direction worthy of research. In the future, we will further study this aspect.

## Data Availability Statement

The original contributions presented in the study are included in the article/supplementary material, further inquiries can be directed to the corresponding author.

## Ethics Statement

Written informed consent was obtained from the individual(s) for the publication of any potentially identifiable images or data included in this article.

## Author Contributions

GB designed the research, analyzed the data, and wrote the manuscript of this study. BY designed the research. LT designed the research and analyzed the data. JS collected the data and contributed to the production of charts. LW analyzed the data and retrieved the document. KY collected the data. YZ collected the data and wrote the manuscript. All authors contributed to the article and approved the submitted version.

## Conflict of Interest

The authors declare that the research was conducted in the absence of any commercial or financial relationships that could be construed as a potential conflict of interest.

## Publisher’s Note

All claims expressed in this article are solely those of the authors and do not necessarily represent those of their affiliated organizations, or those of the publisher, the editors and the reviewers. Any product that may be evaluated in this article, or claim that may be made by its manufacturer, is not guaranteed or endorsed by the publisher.
